# Burden for the infection control programme of a European hospital of getting prepared and treat a patient (PT) with Ebola virus disease (EVD)

**DOI:** 10.1186/2047-2994-4-S1-O14

**Published:** 2015-06-16

**Authors:** A Iten, MN Charaïti, C Ginet, P Brennenstuhl, D Pittet

**Affiliations:** 1 Infection Control, University of Geneva Hospitals (HUG), Geneva, Switzerland; 2 Department of Nursing and Hospital Directory, University of Geneva Hospitals (HUG), Geneva, Switzerland

## Introduction

Managing a patient (PT) with Ebola Virus Disease (EVD) requires a large number of experts from different specialties, including infection control specialists (ICS). In such a situation, the priority task for ICS is to develop recommendations and provide training and supervision of the use of PPE and the management of PT needs and generated waste.

## Objectives

We describe the preparative work (before PT admission) and support (during hospital stay) provided by IPS for the care of a PT suffering from EVD.

## Methods

Data collection to quantify the workload on the IPS team to prepare for, implement and monitor IC procedures undertaken for EVD.

## Results

March to Nov 2014: revision of written recommendations, procedures and checklists to manage EVD from arrival to the airport to hospital discharge. We assigned one IC nurse and one IC physician to this tasks. From 9/2014 to 19/11/2014: information/1 h-training sessions for n=100, 100, 30, and 20 HCW in ICU, adult emergency room (ER), internal medicine and pediatric ER, respectively. In addition, 20 healthcare workers (HCWs) were trained as supervisors. From 19/11 to 6/12/2014, the EVD PT was admitted in a dedicated isolation room in the ICU. During this period, each day, nine ICU nurses (three/shift) were dedicated to PT care & three IC nurses (one/shift in addition to IC physicians) to the supervision of IC practices. HCWs spent a total of 2479 hours in the area dedicated to the EVD PT, who was discharged on 6/12/2014. At time, 15 HCWs proceeded to the decontamination of the dedicated area - cleansing of materials, equipments, and environment used (1 day). In the light of this experience, the recommendations, procedures and checklists were revisited.

## Conclusion

Despite prior long-standing preparative work, the support of an EVD PT requires the participation of a very large number of intensively trained, expert HCW. The burden on the IPS team is huge. Treatment of a EVD PT requires the investment and the contribution of the entire institution.

## Disclosure of interest

None declared.

